# Supplementary material for: Expanding cancer predisposition genes with ultra-rare cancer-exclusive human variations
Source: Sci Rep. 2020 Aug 10;10:13462. doi: 10.1038/s41598-020-70494-0 (PMC7418036; doi:10.1038/s41598-020-70494-0)
Supplement: Supplementary file 1 — Supplementary Supplementary Information 1. [file 41598_2020_70494_MOESM1_ESM.docx]

**Supplementary Figures**

**Expanding cancer predisposition genes with ultra-rare cancer-exclusive human variations**

Roni Rasnic^1*^, Nathan Linial^1^ and Michal Linial^2^

^1^The Rachel and Selim Benin School of Computer Science and Engineering, ^2^Department of Biological Chemistry, Institute of Life Sciences, The Hebrew University of Jerusalem, Jerusalem, Israel

**Supplementary figures S1-S24.** A document containing survival and disease progression estimates for each TCGA cancer type with at least 100 samples. The analyses compare individuals with and without somatic mutation in the 21 novel CPGs.

**Cancer type specific survival and disease/progression estimate**

**Figure S1.** The effect of somatic mutations in the 21 novel CPG candidate on the survival rate of TCGA brain lower grade glioma patients was tested via cBioPortal. The analysis was performed on 507 patients, 19% had somatic mutations in at least one of the novel CPGs. Median overall survival of altered group is 93.2 months. Median overall survival of unaltered group is 78.21 months. **(a)** Overall survival rate estimate. **(b)** Disease / Progression-free estimate.

**brain lower grade glioma**

**a**


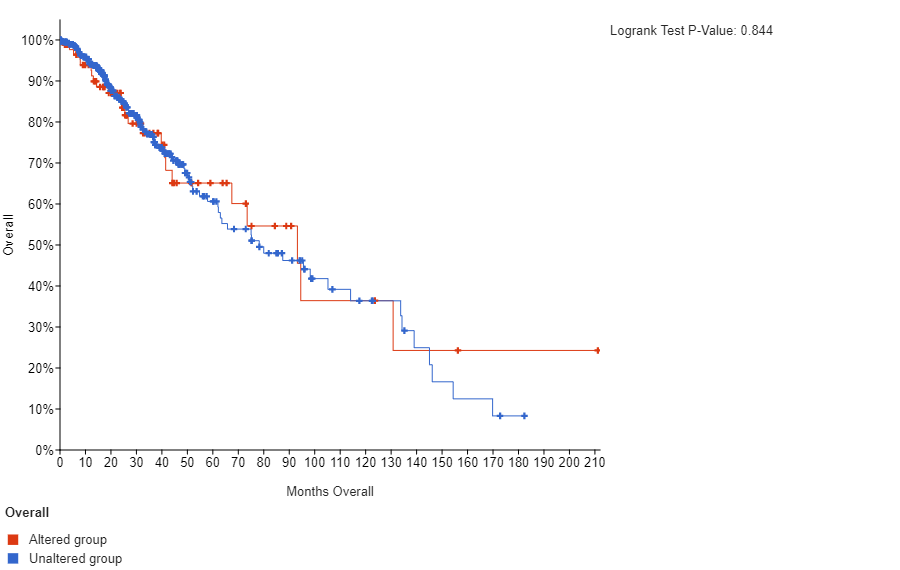


Overall survival

**brain lower grade glioma**

**b**


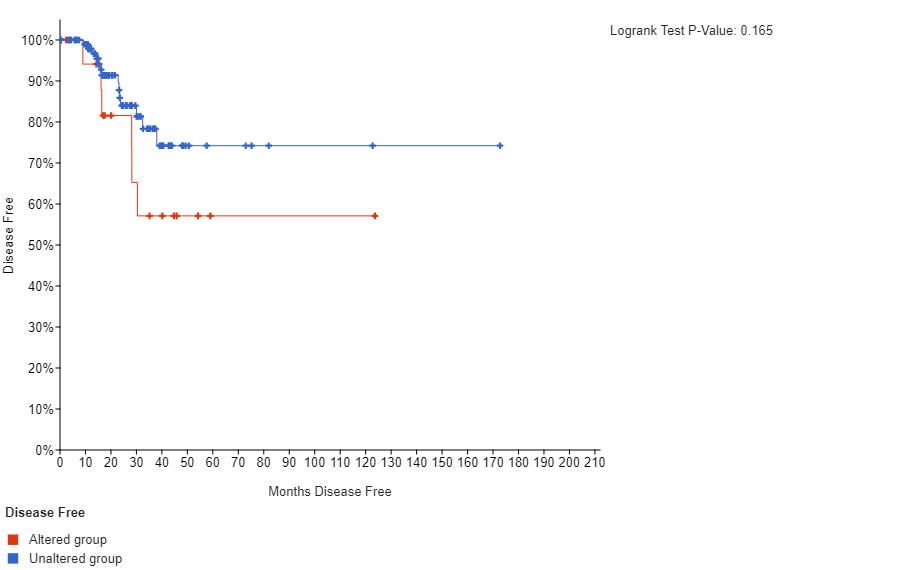


Disease Free survival

**Figure S2.** The effect of somatic mutations in the 21 novel CPG candidate on the survival rate of TCGA bladder urothelial carcinoma patients was tested via cBioPortal. The analysis was performed on 406 patients, 54% had somatic mutations in at least one of the novel CPGs. Median overall survival of altered group is 44.32 months. Median overall survival of unaltered group is 30.94 months. **(a)** Overall survival rate estimate. **(b)** Disease / Progression-free estimate.

**bladder urothelial carcinoma**

**a**


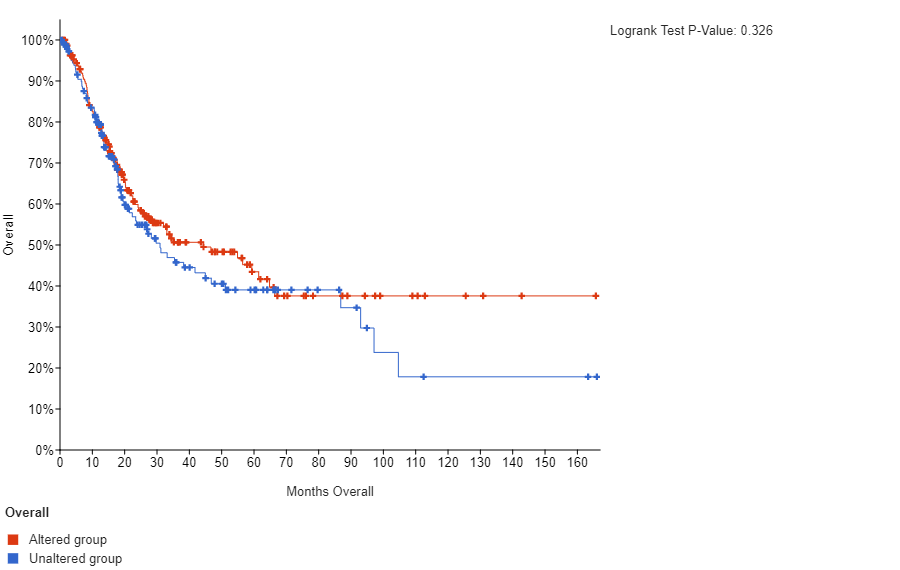


Overall survival

**bladder urothelial carcinoma**

**b**


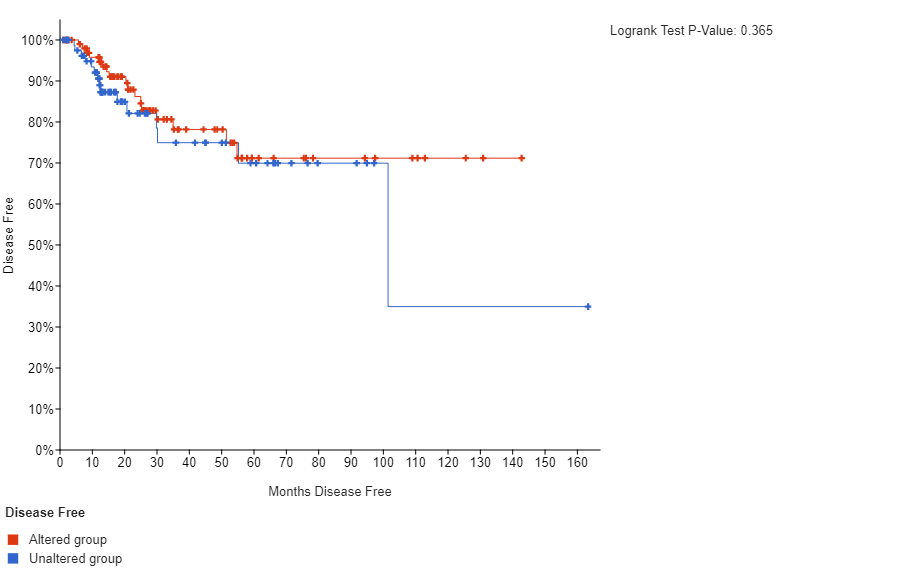


Disease Free survival

**Figure S3.** The effect of somatic mutations in the 21 novel CPG candidate on the survival rate of TCGA breast invasive carcinoma patients was tested via cBioPortal. The analysis was performed on 996 patients, 32% had somatic mutations in at least one of the novel CPGs. Median overall survival of altered group is 113.79 months. Median overall survival of unaltered group is 140.28 months. **(a)** Overall survival rate estimate. **(b)** Disease / Progression-free estimate.

**breast invasive carcinoma**

**a**


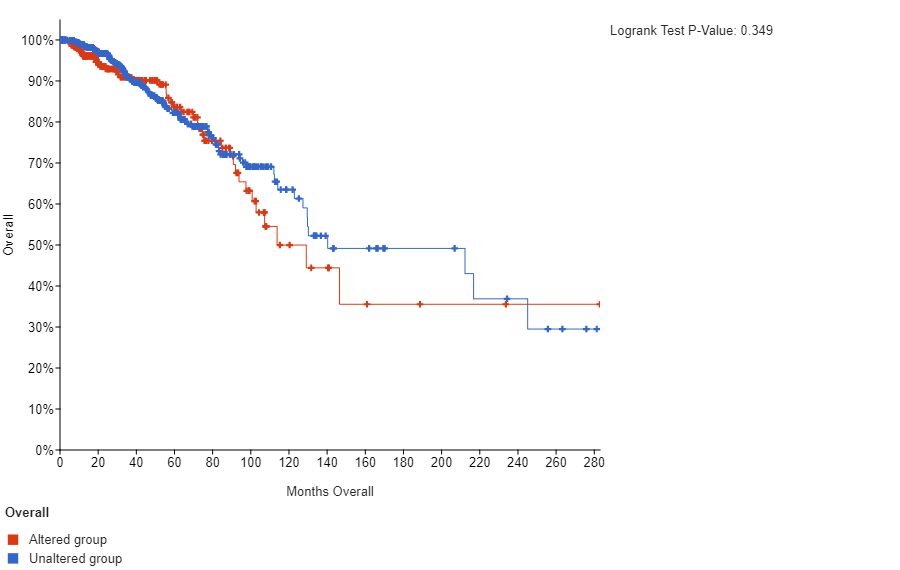


Overall survival

**breast invasive carcinoma**

**b**


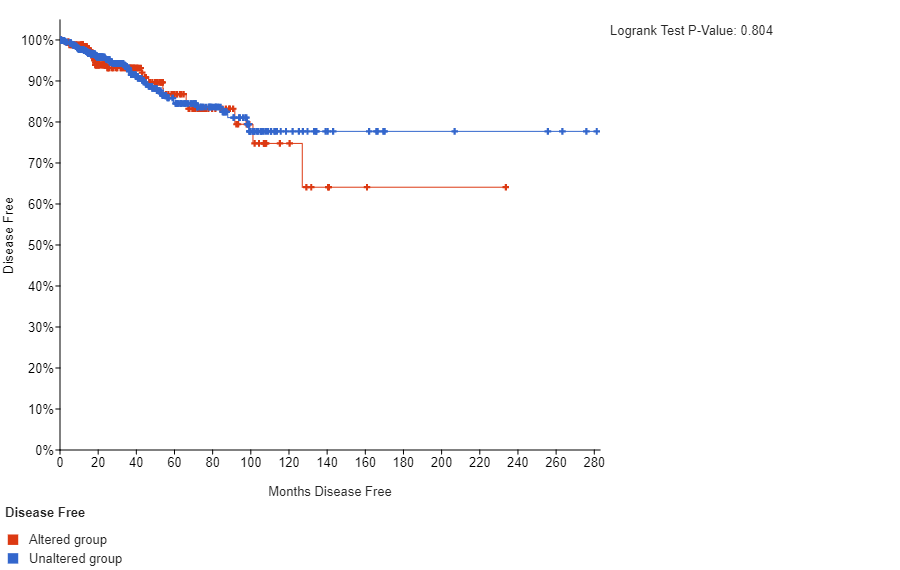


Disease Free survival

**Figure S4.** The effect of somatic mutations in the 21 novel CPG candidate on the survival rate of TCGA cervical squamous cell carcinoma patients was tested via cBioPortal. The analysis was performed on 278 patients, 40% had somatic mutations in at least one of the novel CPGs. Median overall survival of altered group is not available. Median overall survival of unaltered group is 94.95 months. **(a)** Overall survival rate estimate. **(b)** Disease / Progression-free estimate.

**cervical squamous cell carcinoma**

**a**


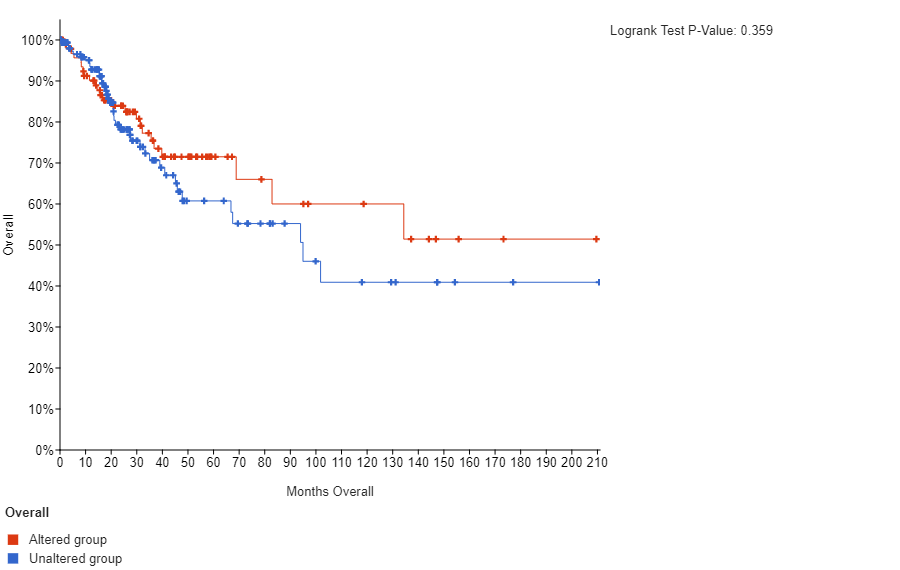


Overall survival

**cervical squamous cell carcinoma**

**b**


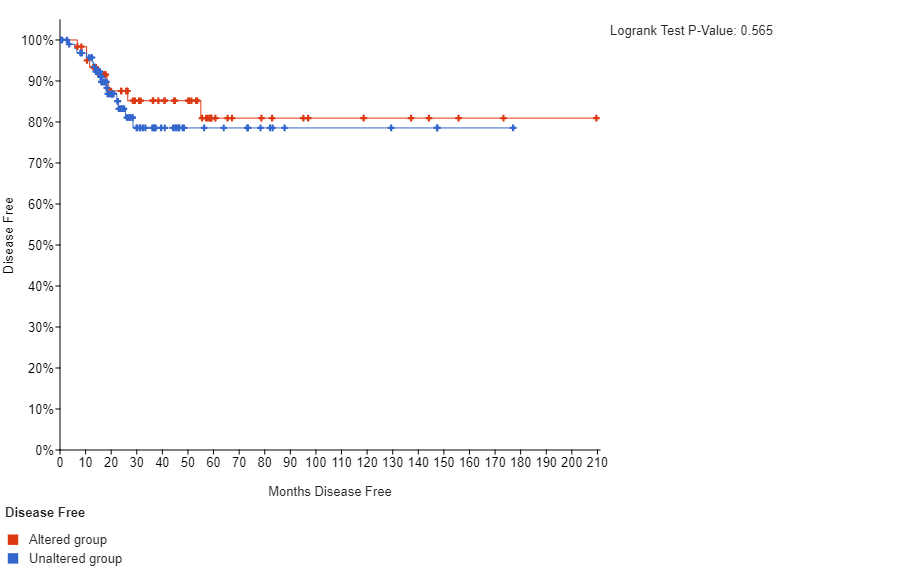


Disease Free survival

**Figure S5.** The effect of somatic mutations in the 21 novel CPG candidate on the survival rate of TCGA colorectal adenocarcinoma patients was tested via cBioPortal. The analysis was performed on 526 patients, 39% had somatic mutations in at least one of the novel CPGs. Median overall survival of altered group is 70.16 months. Median overall survival of unaltered group is 81.37 months. **(a)** Overall survival rate estimate. **(b)** Disease / Progression-free estimate.

**colorectal adenocarcinoma**

**a**


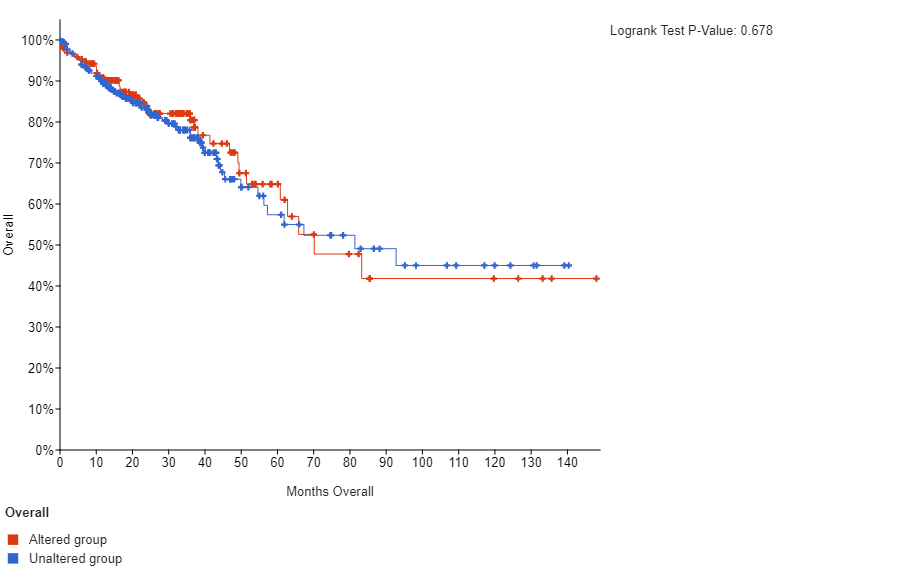


Overall survival

**colorectal adenocarcinoma**

**b**


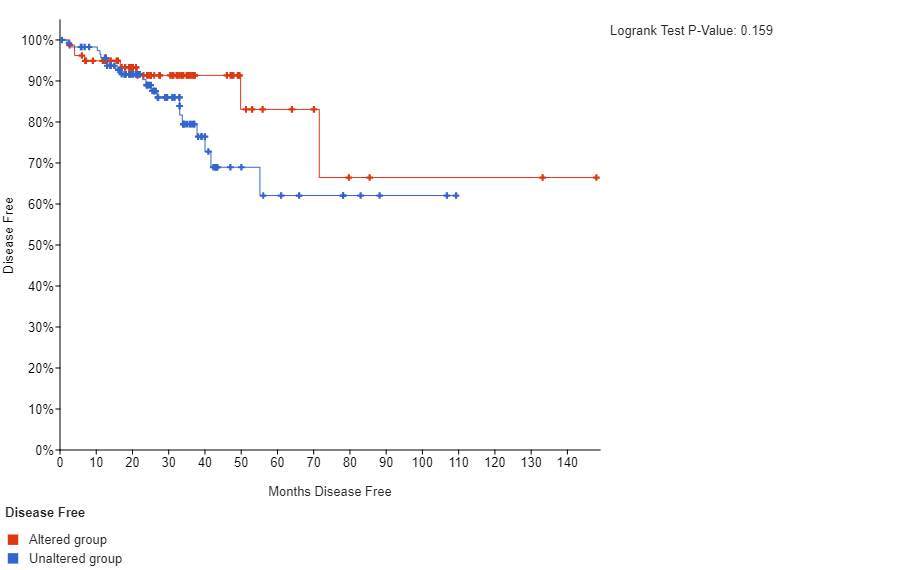


Disease Free survival

**Figure S6.** The effect of somatic mutations in the 21 novel CPG candidate on the survival rate of TCGA esophageal adenocarcinoma patients was tested via cBioPortal. The analysis was performed on 182 patients, 56% had somatic mutations in at least one of the novel CPGs. Median overall survival of altered group is 24 months. Median overall survival of unaltered group is 31.27 months. **(a)** Overall survival rate estimate. **(b)** Disease / Progression-free estimate.

**esophageal adenocarcinoma**

**a**


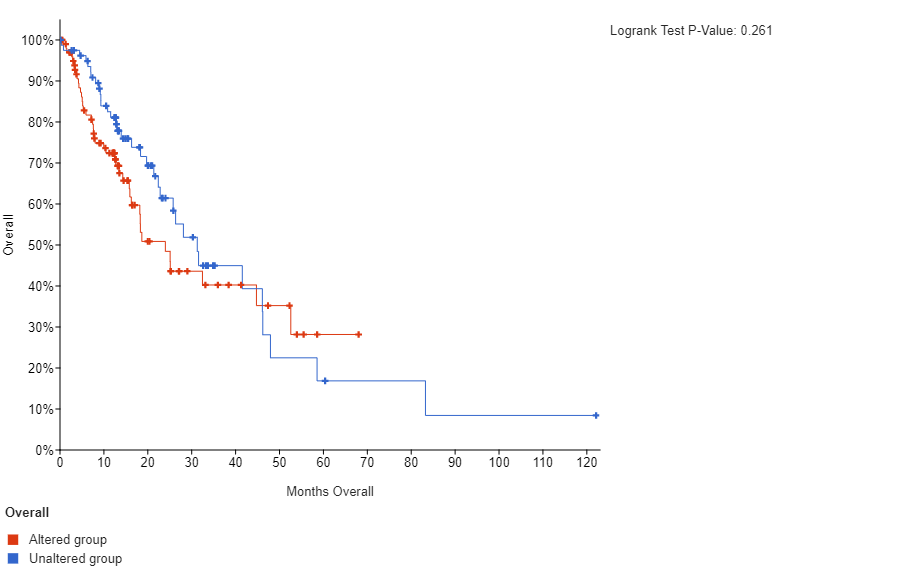


Overall survival

**esophageal adenocarcinoma**

**b**


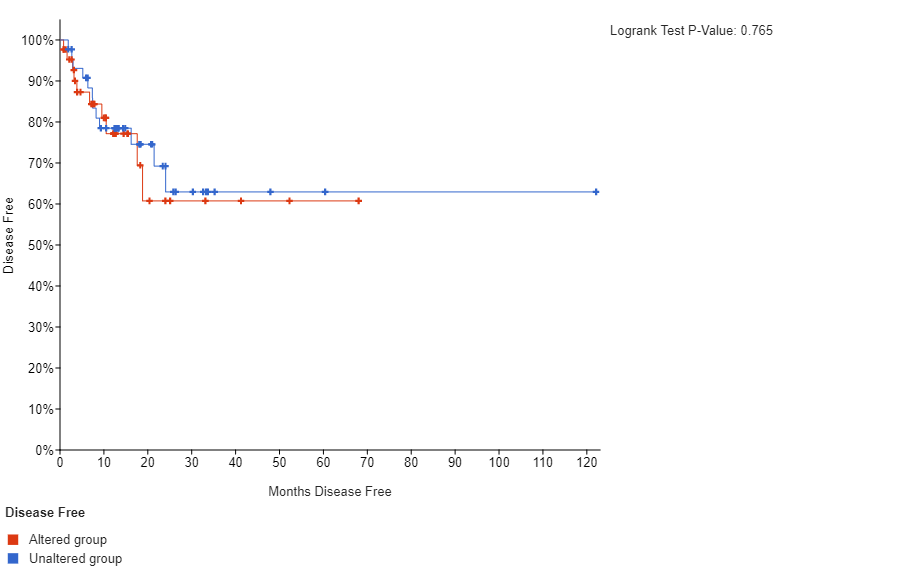


Disease Free survival

**Figure S7.** The effect of somatic mutations in the 21 novel CPG candidate on the survival rate of TCGA glioblastoma multiform patients was tested via cBioPortal. The analysis was performed on 378 patients, 23% had somatic mutations in at least one of the novel CPGs. Median overall survival of altered group is 15.78 months. Median overall survival of unaltered group is 13.78 months. **(a)** Overall survival rate estimate. **(b)** Disease / Progression-free estimate.

**glioblastoma multiform**

**a**


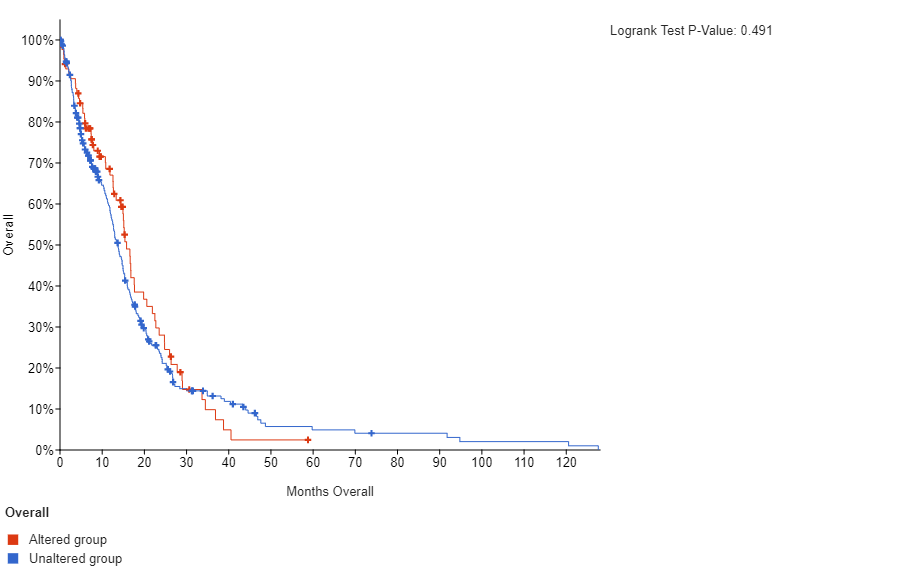


Overall survival

**glioblastoma multiform**

**b**


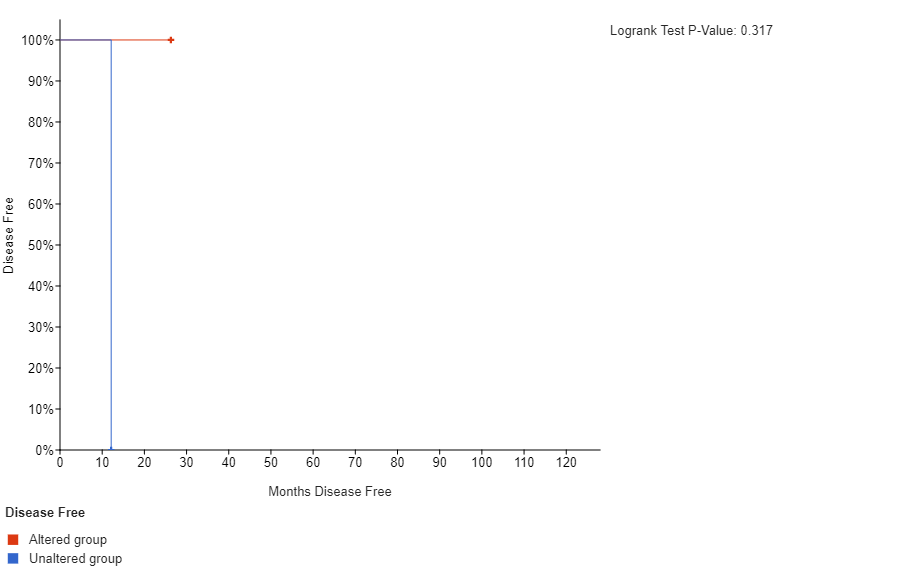


Disease Free survival

**Figure S8.** The effect of somatic mutations in the 21 novel CPG candidate on the survival rate of TCGA head and neck squamous cell carcinoma patients was tested via cBioPortal. The analysis was performed on 496 patients, 38% had somatic mutations in at least one of the novel CPGs. Median overall survival of altered group is 56.48 months. Median overall survival of unaltered group is 57.47 months. **(a)** Overall survival rate estimate. **(b)** Disease / Progression-free estimate.

**head and neck squamous cell carcinoma**

**a**

**head and neck squamous cell carcinoma**


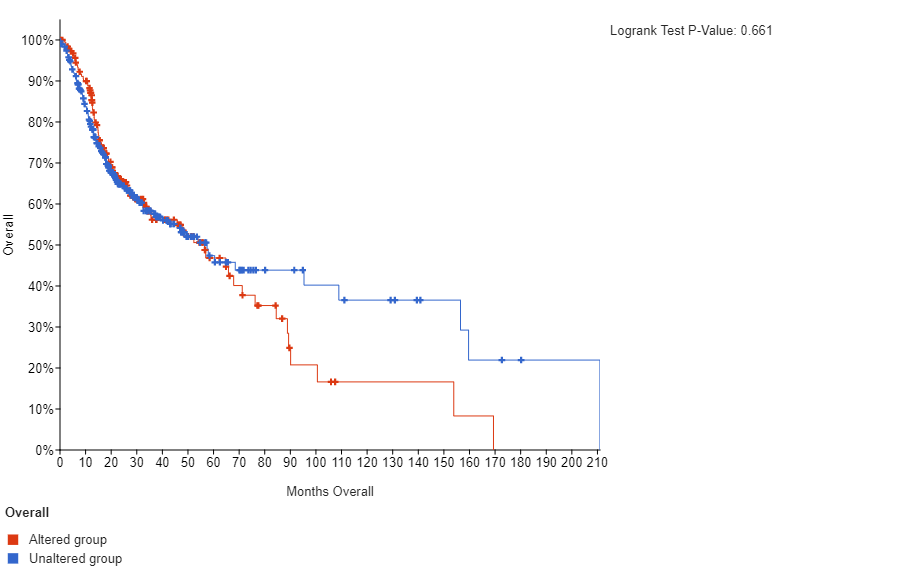


Overall survival

**head and neck squamous cell carcinoma**

**b**


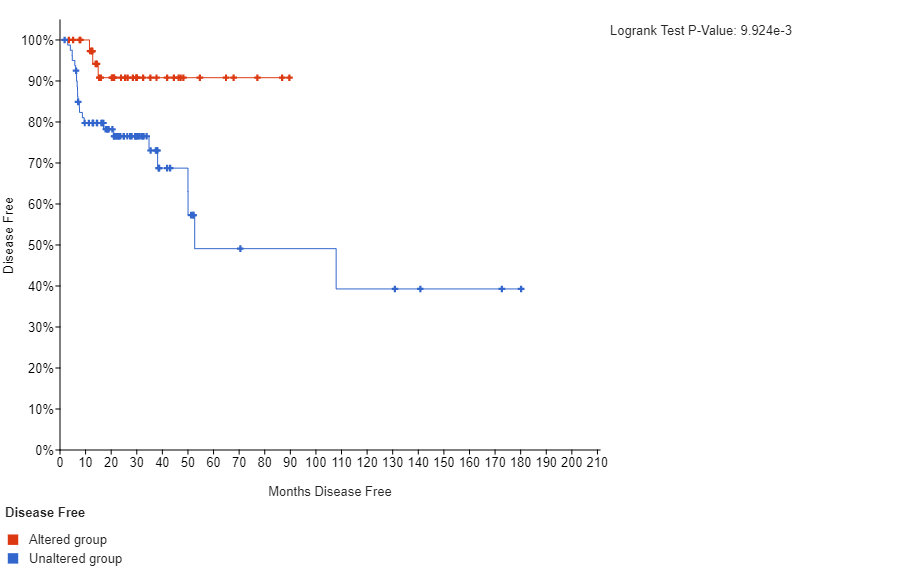


Disease Free survival

**Figure S9.** The effect of somatic mutations in the 21 novel CPG candidate on the survival rate of TCGA kidney renal clear cell carcinoma patients was tested via cBioPortal. The analysis was performed on 354 patients, 21% had somatic mutations in at least one of the novel CPGs. Median overall survival of altered group is 90.87 months. Median overall survival of unaltered group is not available. **(a)** Overall survival rate estimate. **(b)** Disease / Progression-free estimate.

**a**

**kidney renal clear cell carcinoma**


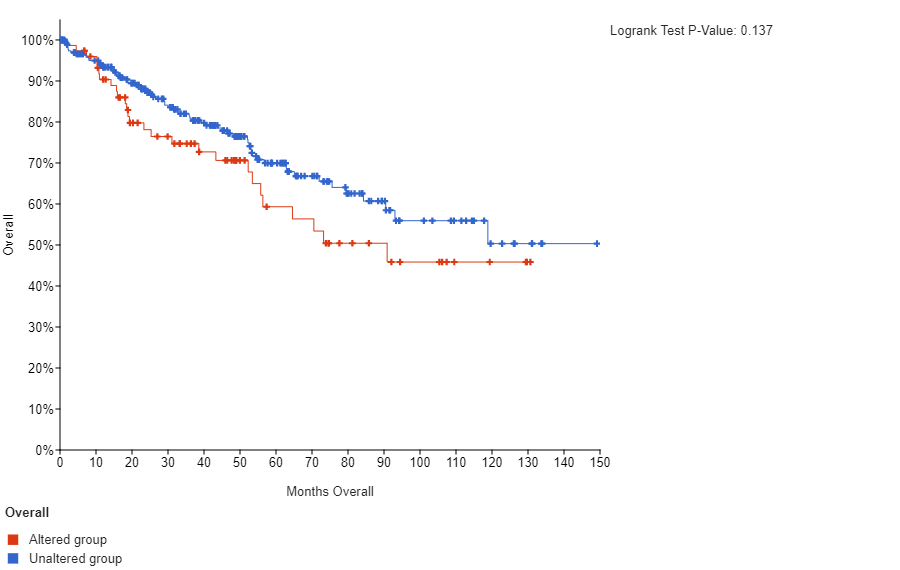


Overall survival

**kidney renal clear cell carcinoma**

**b**


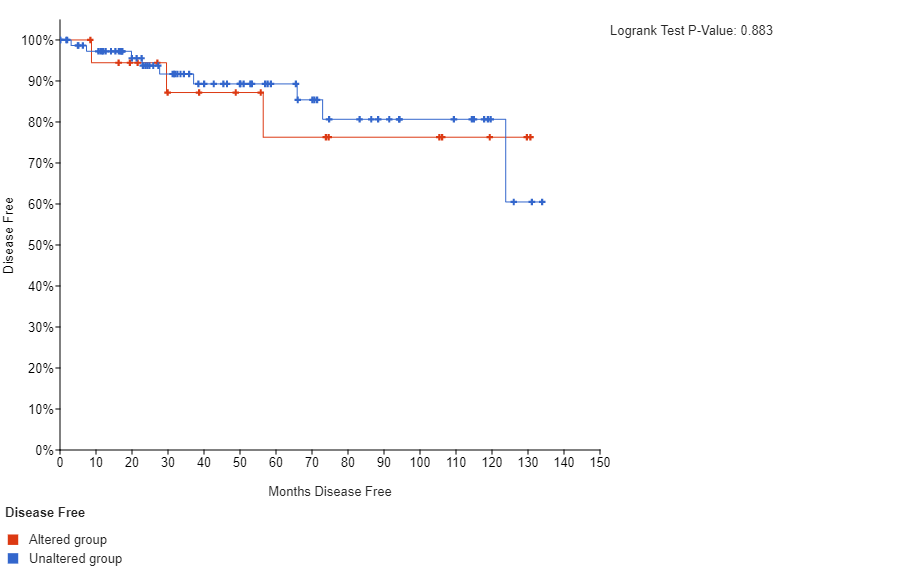


Disease Free survival

**Figure S10.** The effect of somatic mutations in the 21 novel CPG candidate on the survival rate of TCGA kidney renal papillary cell carcinoma patients was tested via cBioPortal. The analysis was performed on 274 patients, 16% had somatic mutations in at least one of the novel CPGs. Median overall survival is not available. **(a)** Overall survival rate estimate. **(b)** Disease / Progression-free estimate.

**kidney renal papillary cell carcinoma**

**a**


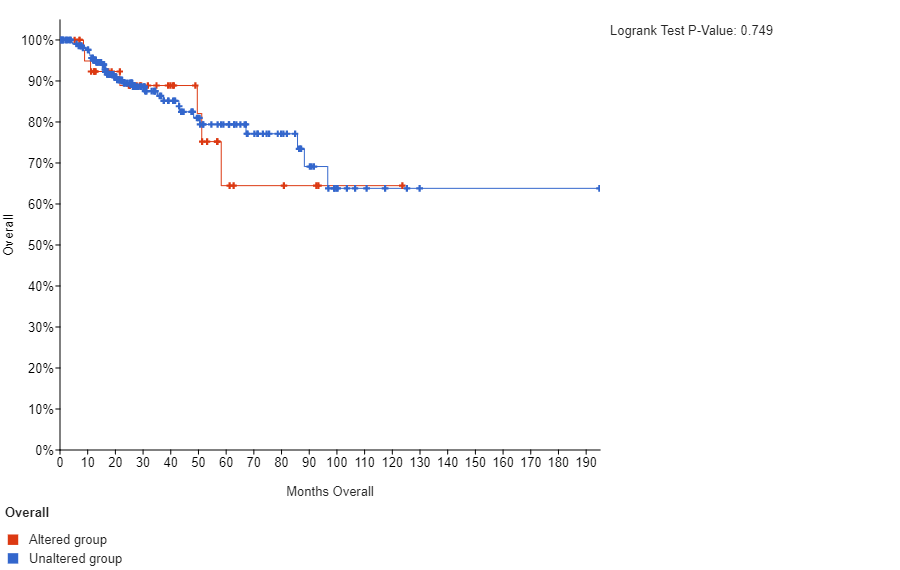


Overall survival

**kidney renal papillary cell carcinoma**

**b**


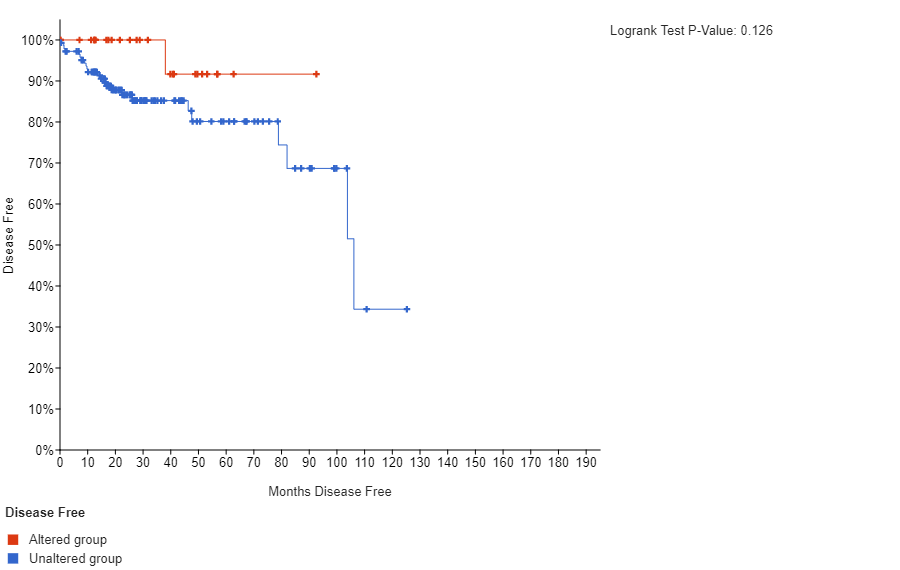


Disease Free survival

**Figure S11.** The effect of somatic mutations in the 21 novel CPG candidate on the survival rate of TCGA liver hepatocellular cell carcinoma patients was tested via cBioPortal. The analysis was performed on 353 patients, 33% had somatic mutations in at least one of the novel CPGs. Median overall survival of altered group is 53.33 months. Median overall survival of unaltered group is 70.06 months. **(a)** Overall survival rate estimate. **(b)** Disease / Progression-free estimate.

**liver hepatocellular cell carcinoma**

**a**

**liver hepatocellular cell carcinoma**


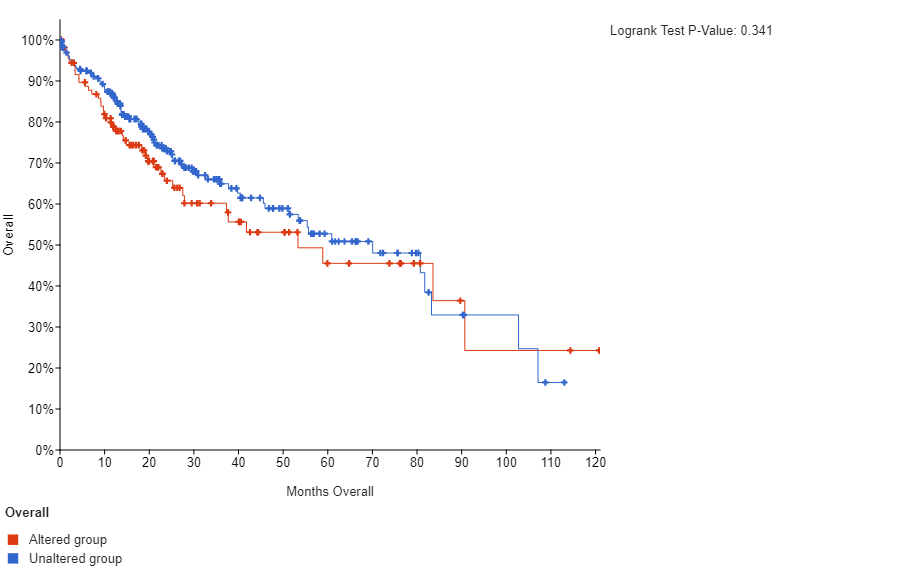


Overall survival

**liver hepatocellular cell carcinoma**

**b**


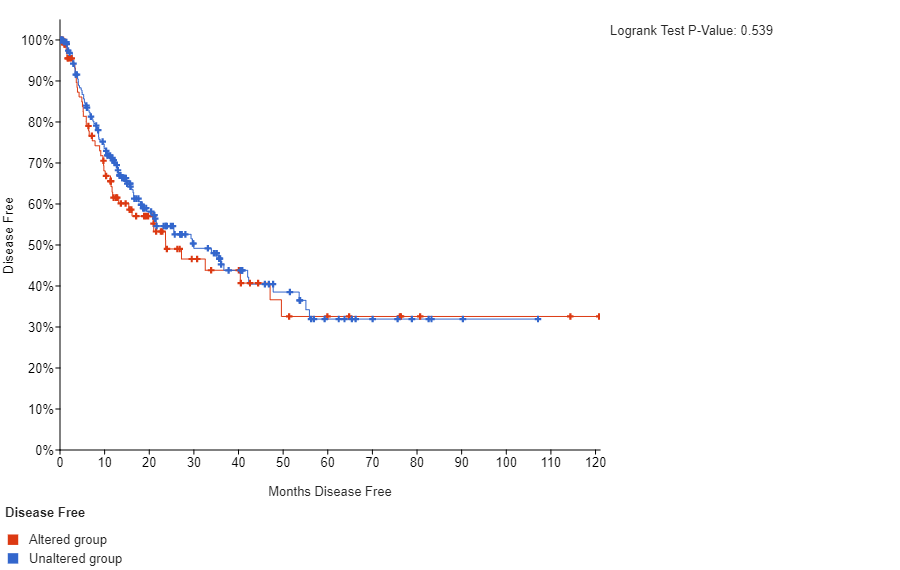


Disease Free survival

**Figure S12.** The effect of somatic mutations in the 21 novel CPG candidate on the survival rate of TCGA lung adenocarcinoma patients was tested via cBioPortal. The analysis was performed on 507 patients, 57% had somatic mutations in at least one of the novel CPGs. Median overall survival of altered group is 49.05 months. Median overall survival of unaltered group is 52.6 months. **(a)** Overall survival rate estimate. **(b)** Disease / Progression-free estimate.

**a**

**lung adenocarcinoma**


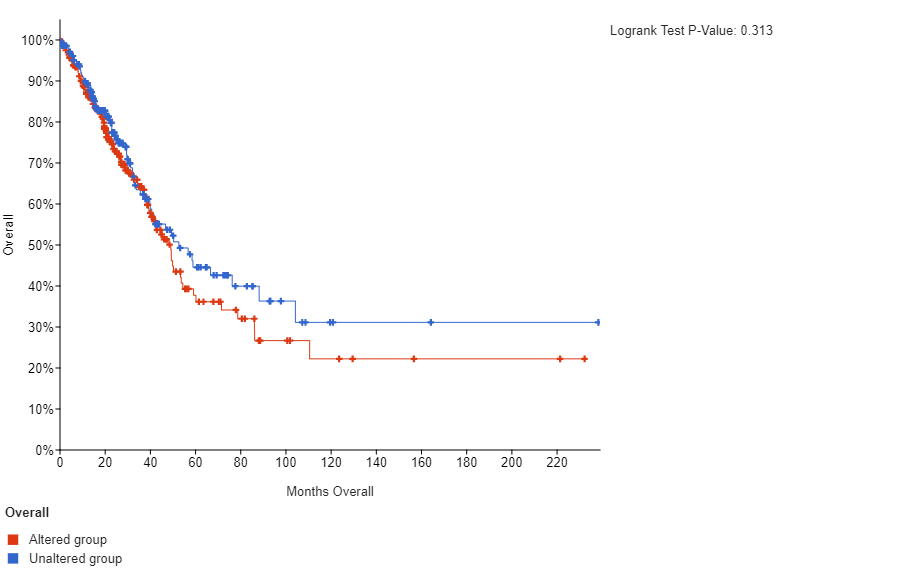


Overall survival

**lung adenocarcinoma**

**b**


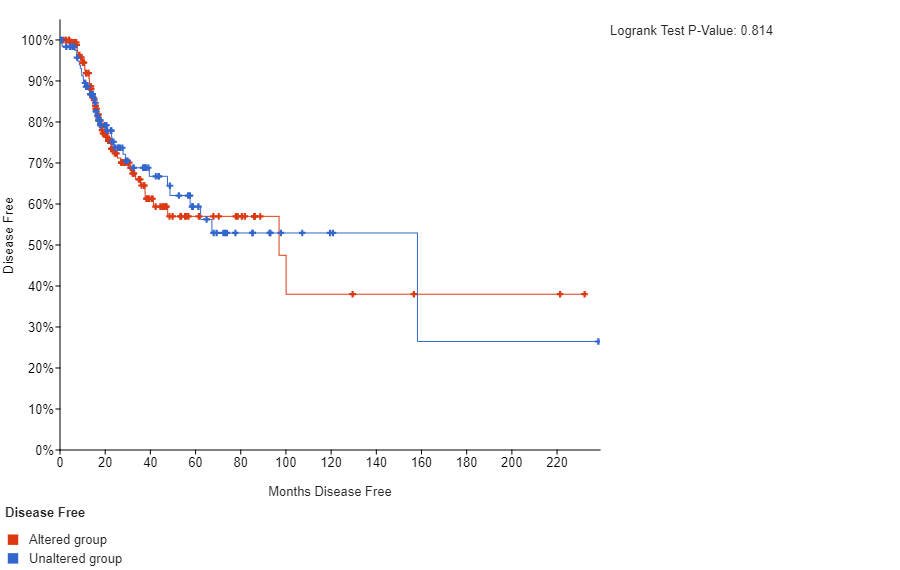


Disease Free survival

**Figure S13.** The effect of somatic mutations in the 21 novel CPG candidate on the survival rate of TCGA lung squamous cell carcinoma patients was tested via cBioPortal. The analysis was performed on 469 patients, 62% had somatic mutations in at least one of the novel CPGs. Median overall survival of altered group is 54.44 months. Median overall survival of unaltered group is 57.07 months. **(a)** Overall survival rate estimate. **(a)** Overall survival rate estimate. **(b)** Disease / Progression-free estimate.

**lung squamous cell carcinoma**

**a**


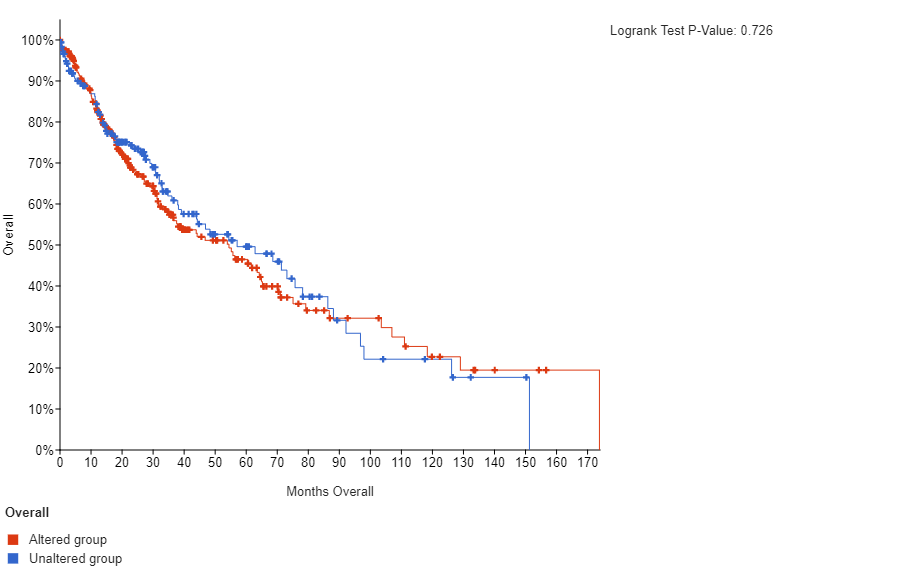


Overall survival

**lung squamous cell carcinoma**

**b**


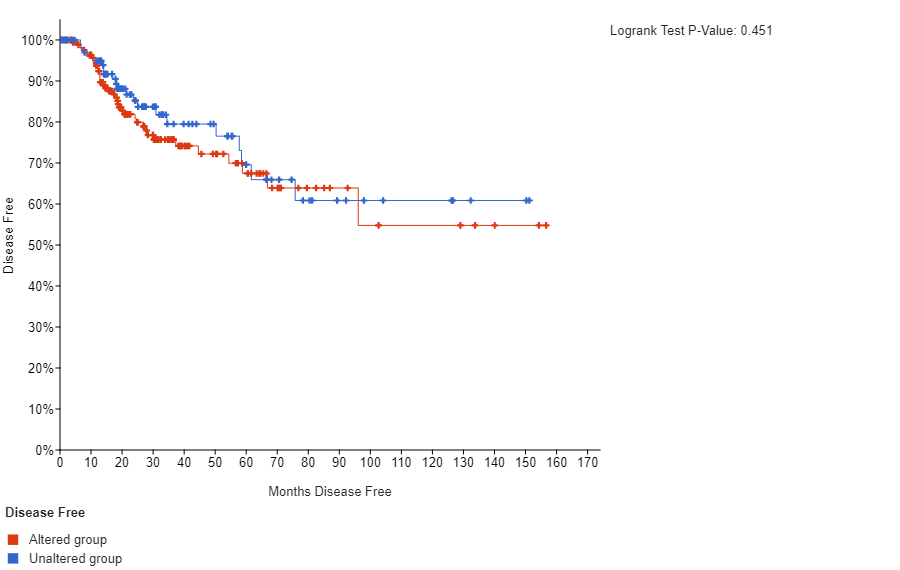


Disease Free survival

**Figure S14.** The effect of somatic mutations in the 21 novel CPG candidate on the overall survival rate estimate of TCGA skin cutaneous melanoma patients was tested via cBioPortal. The analysis was performed on 363 patients, 77% had somatic mutations in at least one of the novel CPGs. Median overall survival of altered group is 98.4 months. Median overall survival of unaltered group is 63.35 months.


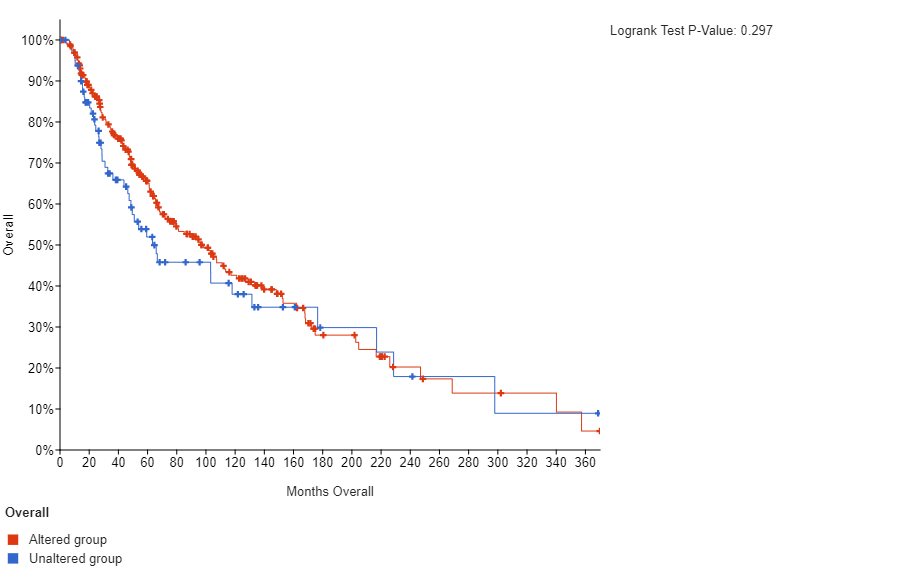


Overall survival

**skin cutaneous melanoma**

**Figure S15.** The effect of somatic mutations in the 21 novel CPG candidate on the overall survival rate estimate of TCGA acute myeloid leukemia patients was tested via cBioPortal. The analysis was performed on 190 patients, 9% had somatic mutations in at least one of the novel CPGs. Median overall survival of altered group is 17 months. Median overall survival of unaltered group is 15.02 months.


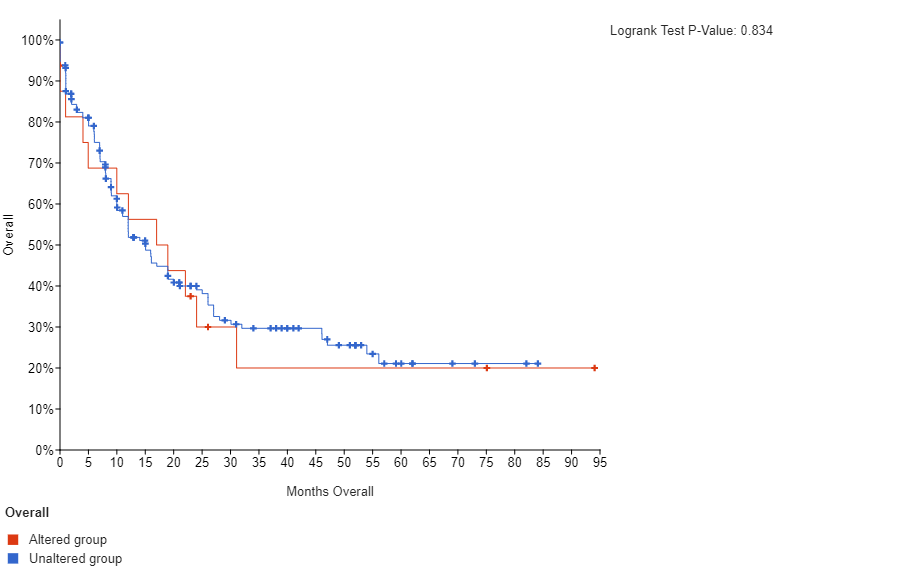


Overall survival

**acute myeloid leukemia**

**Figure S16.** The effect of somatic mutations in the 21 novel CPG candidate on the survival rate of TCGA ovarian serous cystadenocarcinoma patients was tested via cBioPortal. The analysis was performed on 398 patients, 52% had somatic mutations in at least one of the novel CPGs. Median overall survival of altered group is 45.14 months. Median overall survival of unaltered group is 44.51 months. **(a)** Overall survival rate estimate. **(b)** Disease / Progression-free estimate.

**ovarian serous cystadenocarcinoma**

**a**


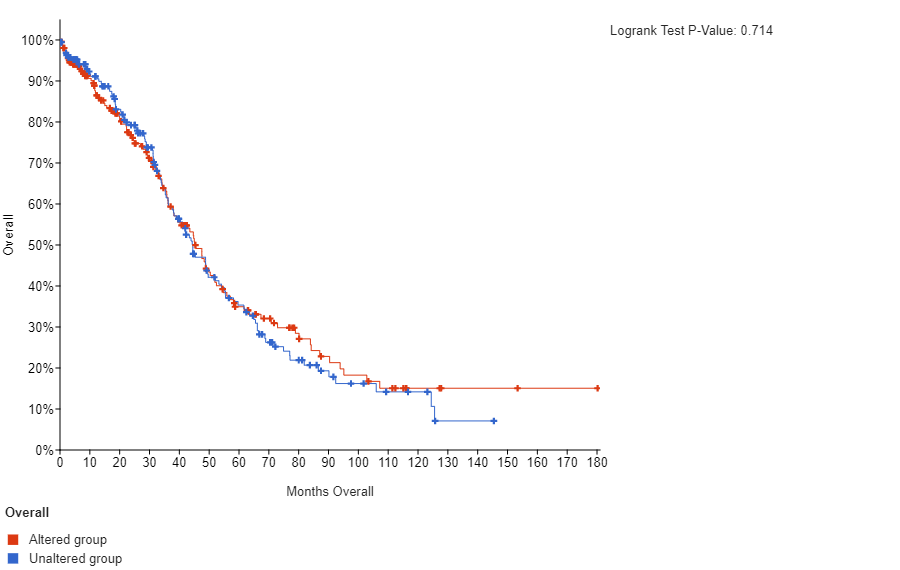


Overall survival

**ovarian serous cystadenocarcinoma**

**b**


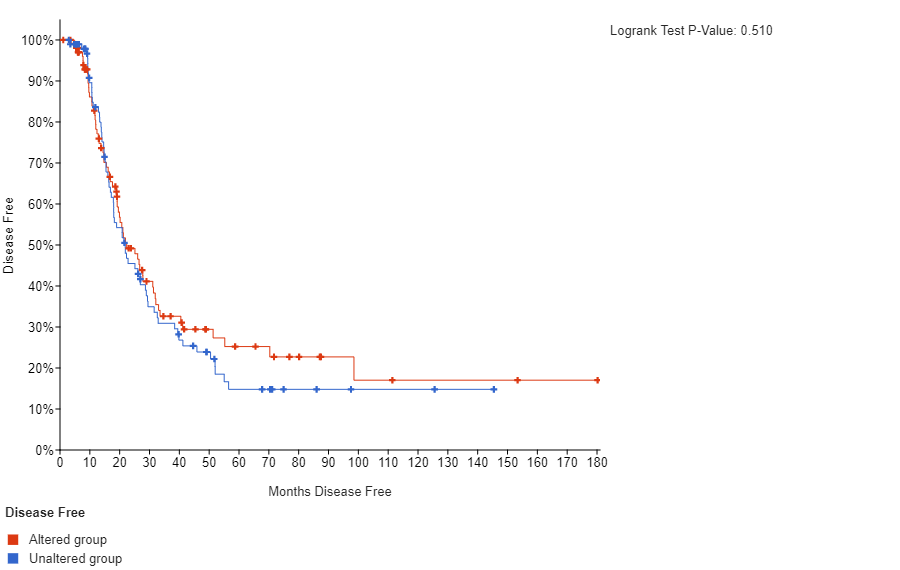


Disease Free survival

**Figure S17.** The effect of somatic mutations in the 21 novel CPG candidate on the survival rate of TCGA pancreatic adenocarcinoma patients was tested via cBioPortal. The analysis was performed on 175 patients, 16% had somatic mutations in at least one of the novel CPGs. Median overall survival of altered group is 20.35 months. Median overall survival of unaltered group is 19.96 months. **(a)** Overall survival rate estimate. **(b)** Disease / Progression-free estimate.

**pancreatic adenocarcinoma**

**a**


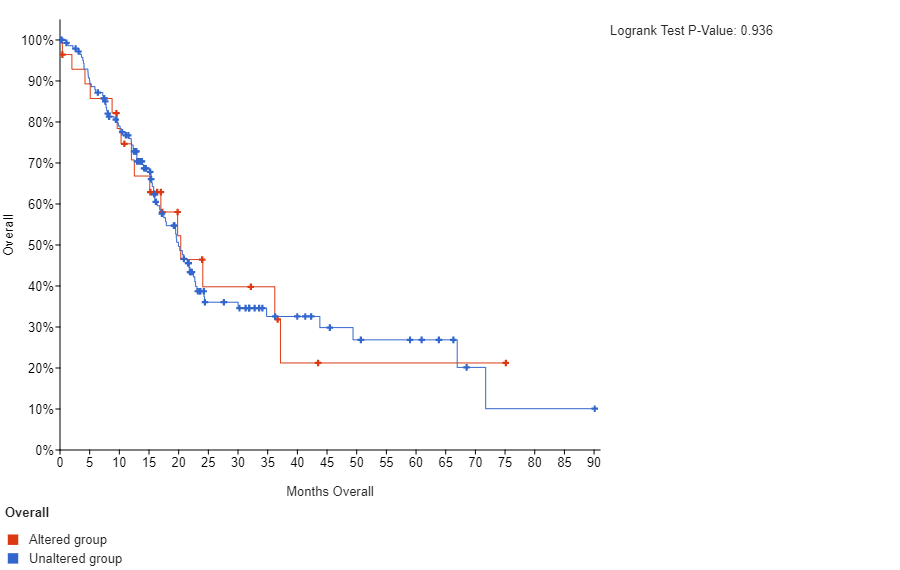


Overall survival

**pancreatic adenocarcinoma**

**b**


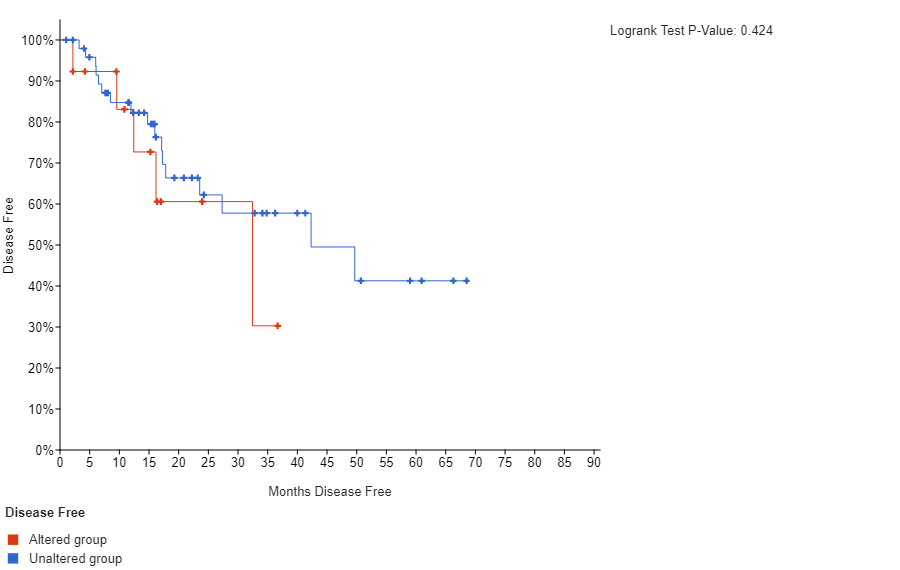


Disease Free survival

**Figure S18.** The effect of somatic mutations in the 21 novel CPG candidate on the survival rate of TCGA prostate adenocarcinoma patients was tested via cBioPortal. The analysis was performed on 489 patients, 22% had somatic mutations in at least one of the novel CPGs. Median overall survival is not available. **(a)** Overall survival rate estimate. **(b)** Disease / Progression-free estimate.

**prostate adenocarcinoma**

**a**


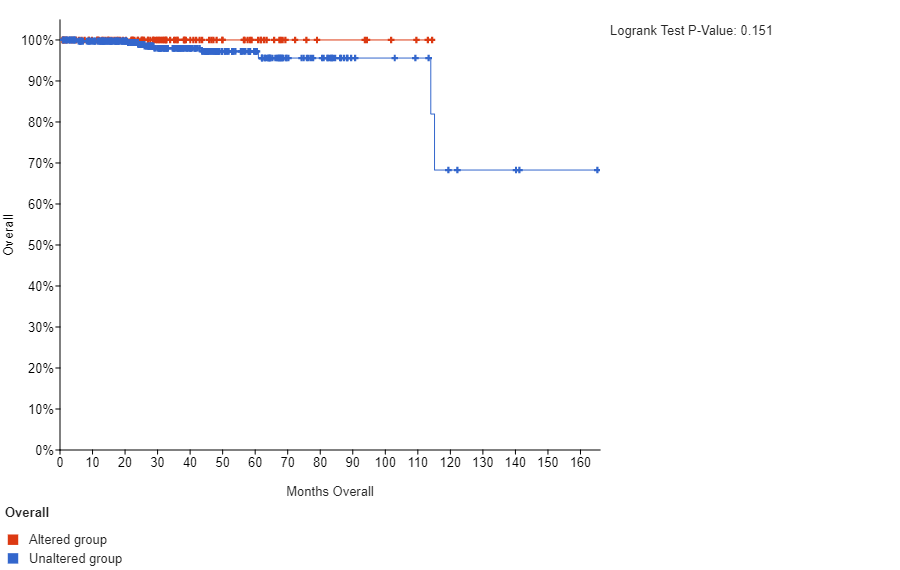


Overall survival

**prostate adenocarcinoma**

**b**


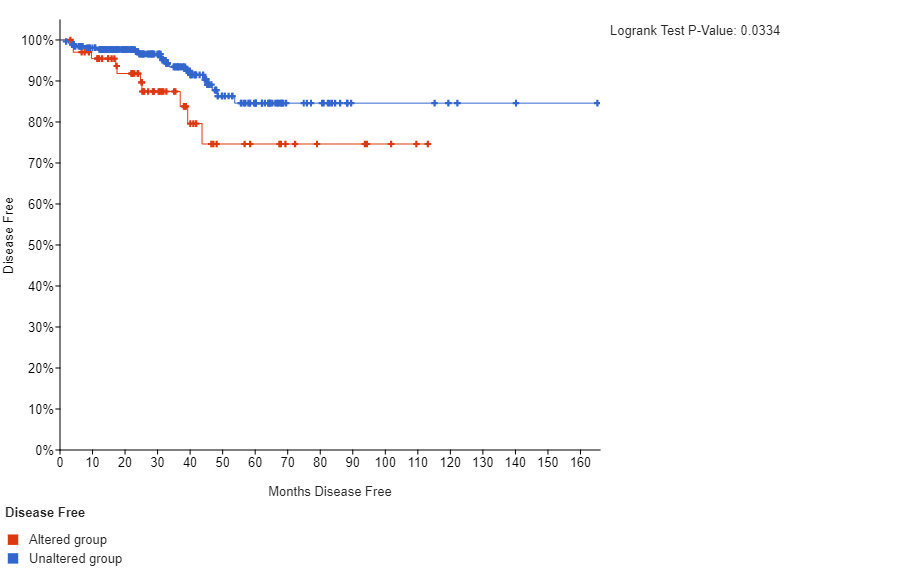


Disease Free survival

**Figure S19.** The effect of somatic mutations in the 21 novel CPG candidate on the survival rate of TCGA sarcoma patients was tested via cBioPortal. The analysis was performed on 253 patients, 49% had somatic mutations in at least one of the novel CPGs. Median overall survival of altered group is 81.01 months. Median overall survival of unaltered group is 63.81 months. **(a)** Overall survival rate estimate. **(b)** Disease / Progression-free estimate.

**sarcoma**

**a**


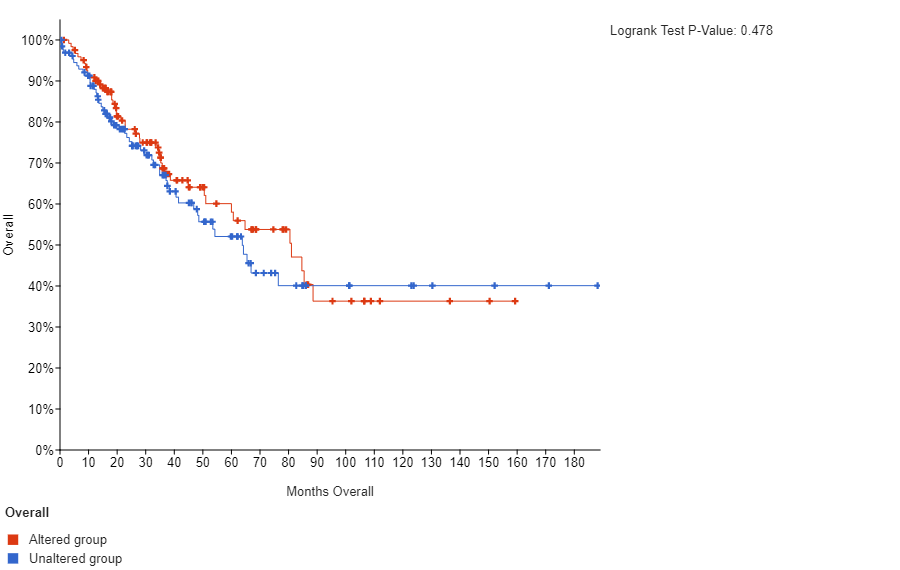


Overall survival

**sarcoma**

**b**


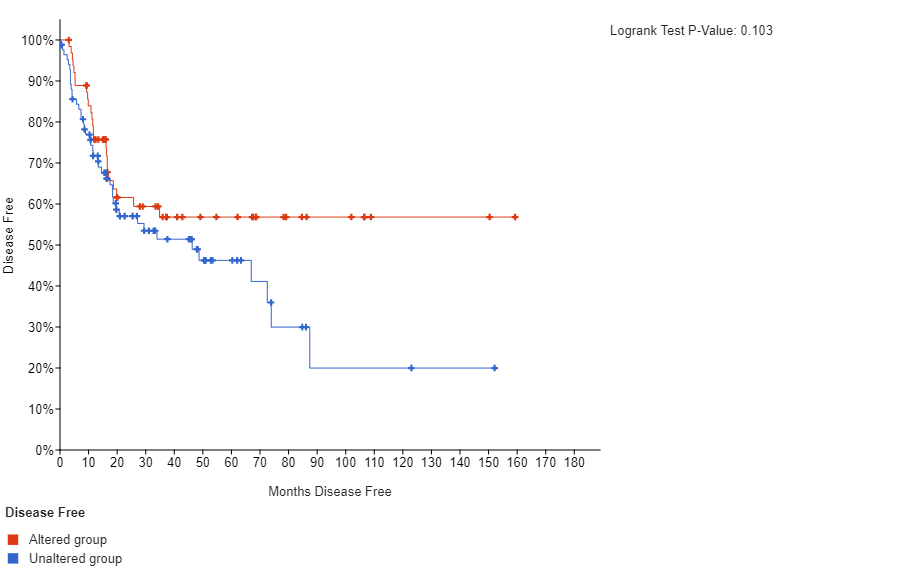


Disease Free survival

**Figure S20.** The effect of somatic mutations in the 21 novel CPG candidate on the survival rate of TCGA stomach adenocarcinoma patients was tested via cBioPortal. The analysis was performed on 434 patients, 55% had somatic mutations in at least one of the novel CPGs. Median overall survival of altered group is 30.9 months. Median overall survival of unaltered group is 42.54 months. **(a)** Overall survival rate estimate. **(b)** Disease / Progression-free estimate.

**stomach adenocarcinoma**

**a**


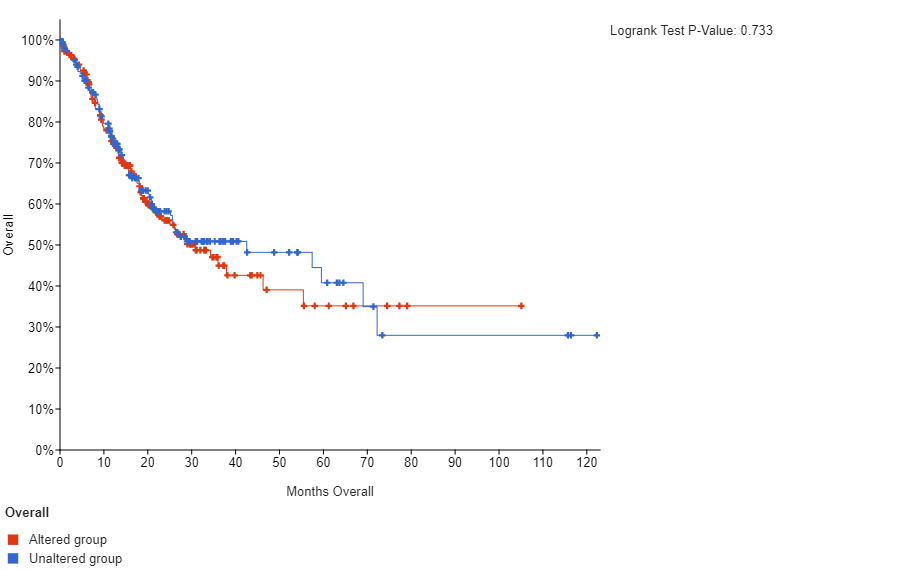


Overall survival

**stomach adenocarcinoma**

**b**


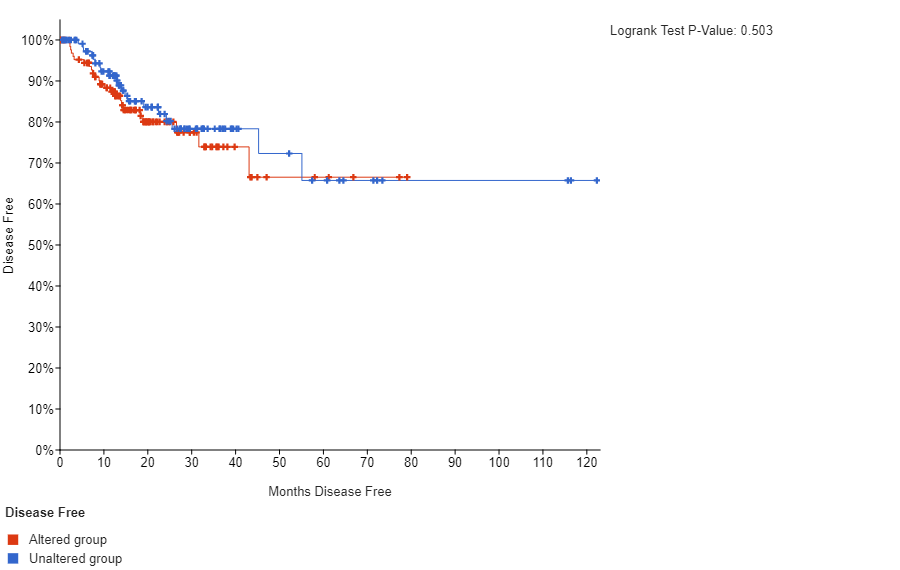


Disease Free survival

**Figure S21.** The effect of somatic mutations in the 21 novel CPG candidate on the survival rate of TCGA testicular germ cells patients was tested via cBioPortal. The analysis was performed on 144 patients, 13% had somatic mutations in at least one of the novel CPGs. Median overall survival is not available. **(a)** Overall survival rate estimate. **(b)** Disease / Progression-free estimate.

**testicular germ cells**

**a**


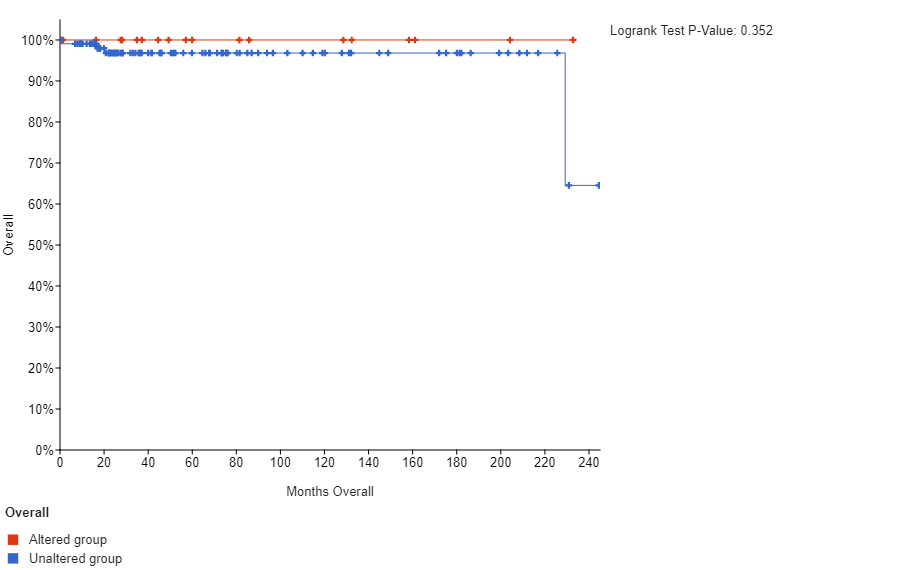


Overall survival

**testicular germ cells**

**b**


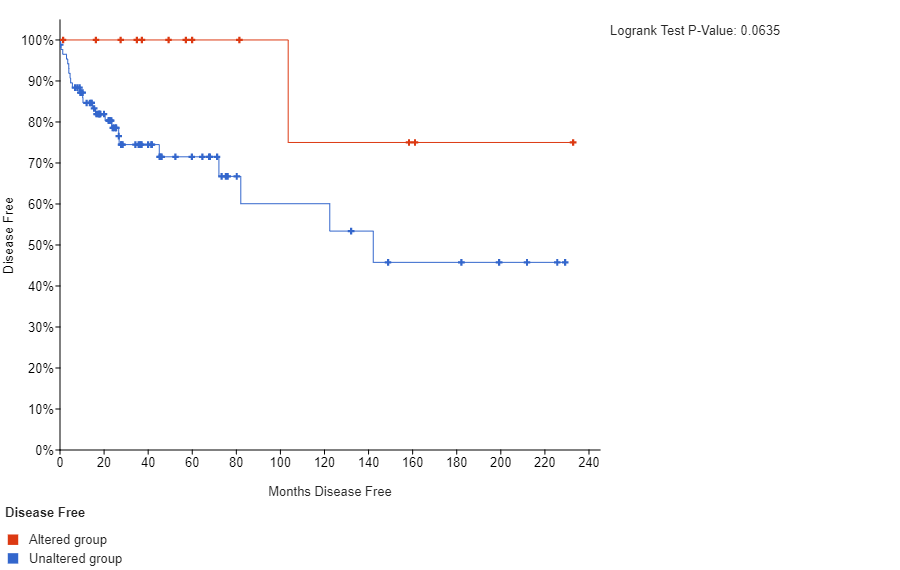


Disease Free survival

**Figure S22.** The effect of somatic mutations in the 21 novel CPG candidate on the overall survival rate estimate of TCGA thymoma patients was tested via cBioPortal. The analysis was performed on 123 patients, 13% had somatic mutations in at least one of the novel CPGs. Median overall survival of altered group is 95.67 months. Median overall survival of unaltered group is not available.

**thymoma**


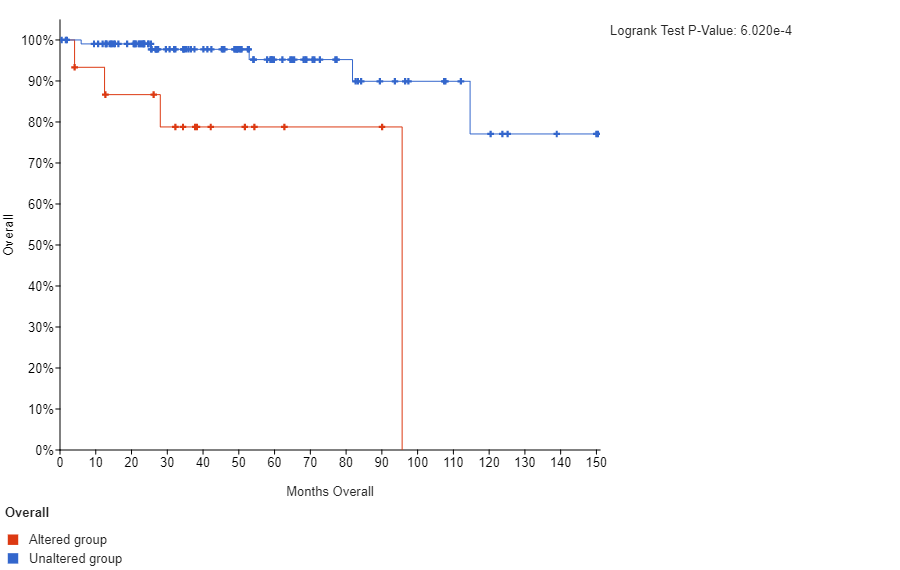


Overall survival

**Figure S23.** The effect of somatic mutations in the 21 novel CPG candidate on the survival rate of TCGA thyroid carcinoma patients was tested via cBioPortal. The analysis was performed on 482 patients, 7% had somatic mutations in at least one of the novel CPGs. Median overall survival is not available. **(a)** Overall survival rate estimate. **(b)** Disease / Progression-free estimate.

**thyroid carcinoma**

**a**


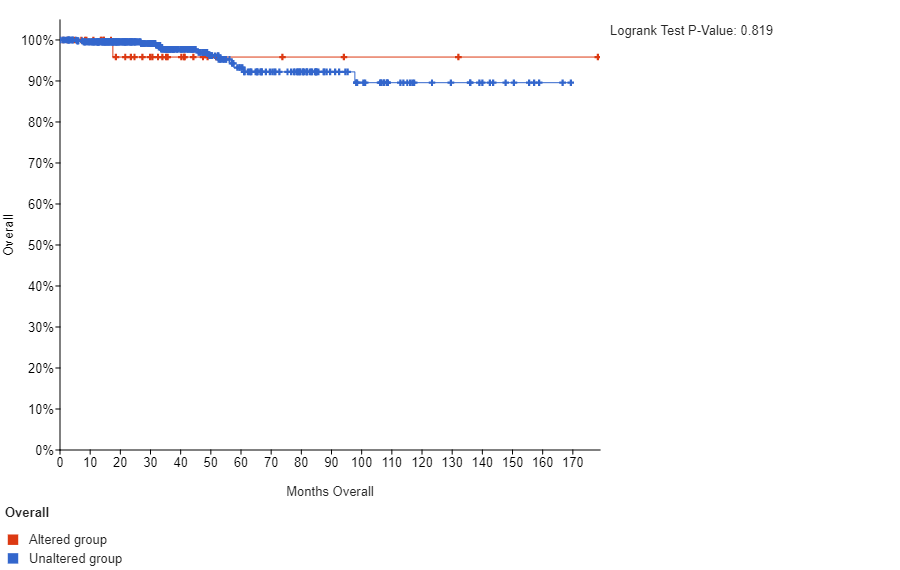


Overall survival

**thyroid carcinoma**

**b**


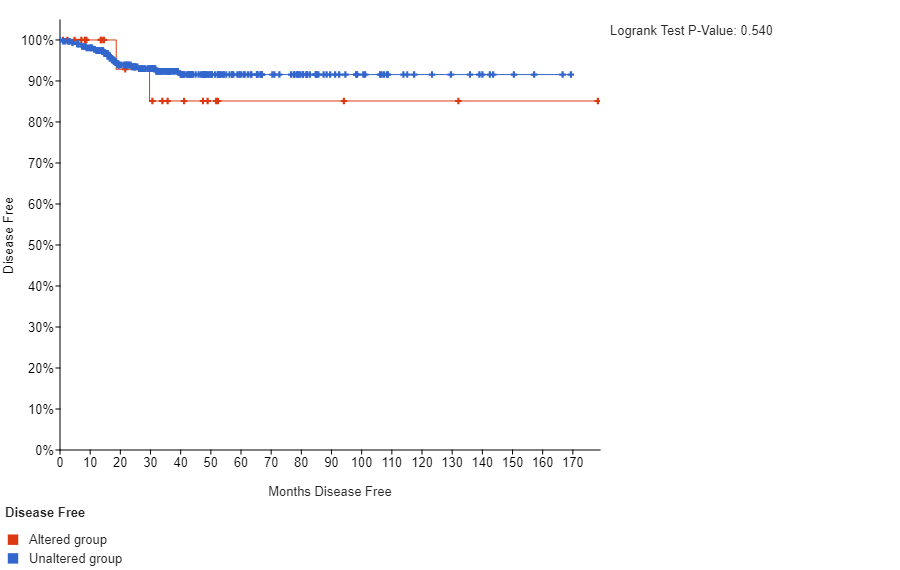


Disease Free survival

**Figure S24.** The effect of somatic mutations in the 21 novel CPG candidate on the survival rate of TCGA uterine corpus endometrial carcinoma patients was tested via cBioPortal. The analysis was performed on 509 patients, 53% had somatic mutations in at least one of the novel CPGs. Median overall survival of altered group is 110.1 months. Median overall survival of unaltered group is not available. **(a)** Overall survival rate estimate. **(b)** Disease / Progression-free estimate.

**uterine corpus endometrial carcinoma**

**a**


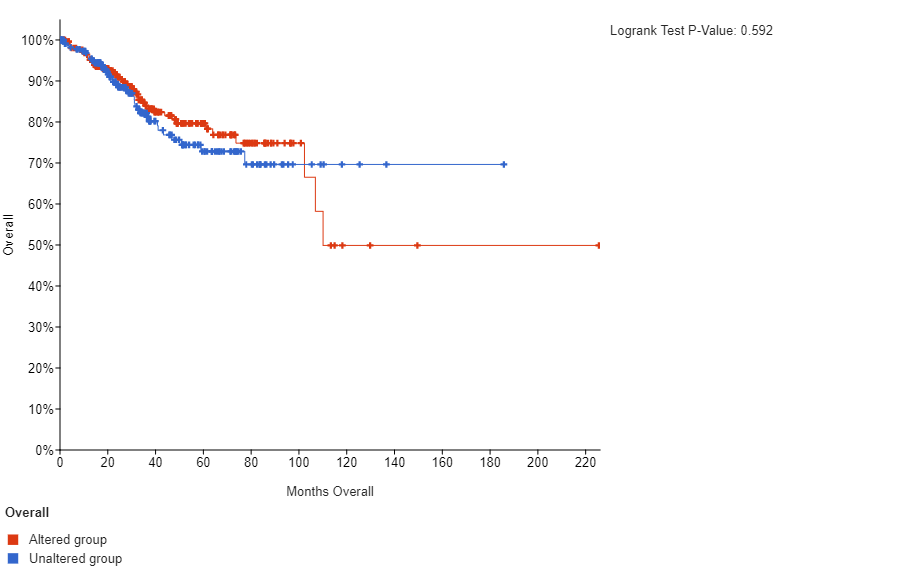


Overall survival

**uterine corpus endometrial carcinoma**

**b**


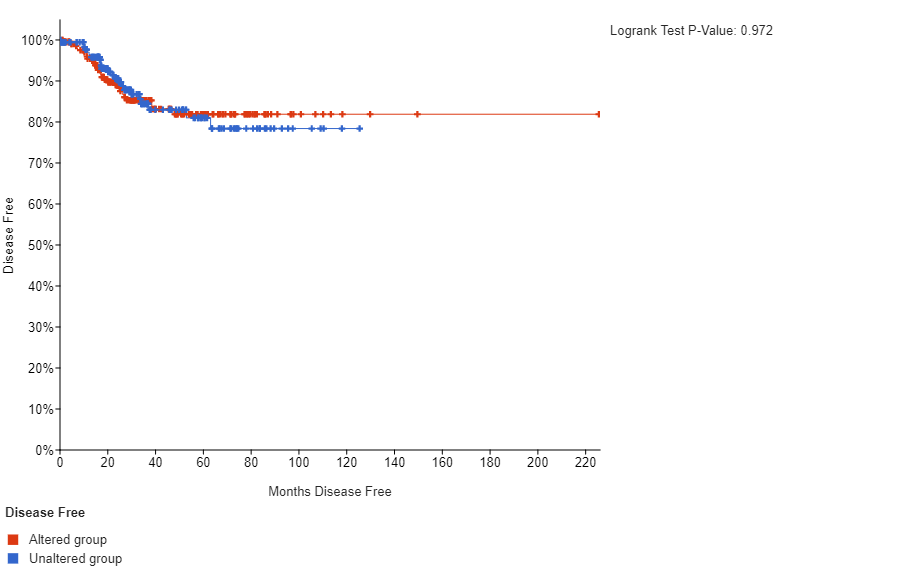


Disease Free survival
